# Targeted Therapies in Infantile Hemangiomas and Vascular Malformations: From β‐Blockers to PI3K/AKT/mTOR Inhibitors

**DOI:** 10.1111/jcmm.71103

**Published:** 2026-04-02

**Authors:** Hubert Arasiewicz, Michal Dec

**Affiliations:** ^1^ Clinical Department of Pediatric Dermatology and Vascular Anomalies, Faculty of Medical Sciences in Katowice, Medical University of Silesia, John Paul II Children's and Family Health Center Sosnowiec Silesian Voivodeship Poland

**Keywords:** infantile hemangioma, PI3K/AKT/mTOR, precision medicine, RAS/MAPK, targeted therapy, vascular malformations

## Abstract

Vascular tumours and malformations encompass infantile hemangiomas (IHs) and genetically driven vascular malformations with distinct natural histories and therapeutic vulnerabilities. The discovery that the non‐selective beta‐blocker propranolol induces rapid regression of proliferating IHs established the first widely adopted systemic pharmacologic therapy in vascular anomaly care and provided a clinical proof‐of‐concept that targeting lesion‐specific endothelial biology can alter disease course. In parallel, recurrent somatic variants affecting PI3K/AKT/mTOR (e.g., PIK3CA, TEK/TIE2, AKT1) and RAS/MAPK (e.g., KRAS, NRAS) signalling have reframed many malformations as mosaic disorders amenable to targeted inhibition with agents such as sirolimus, alpelisib, AKT inhibitors and MEK inhibitors. This review synthesizes translational mechanisms, clinical evidence and safety considerations for beta‐blockers and emerging targeted therapies, emphasizing lesion phenotype, timing of intervention and molecular stratification as determinants of response. We highlight current limitations, including toxicity, durability and pathway escape, and outline future directions for precision therapy and genotype‐guided trial design in vascular anomalies.

## 1 Introduction

Vascular tumours and malformations (VTMs) comprise a biologically heterogeneous group of endothelial disorders, including infantile hemangiomas (IHs) and vascular malformations with distinct clinical courses and therapeutic responsiveness. IHs are benign vascular tumours characterized by a proliferative phase in early infancy followed by spontaneous involution, whereas vascular malformations are congenital lesions that persist throughout life and often progress with growth [1, 2]. IHs are the most common vascular tumours of infancy, with a pooled global prevalence of approximately 2.8% and a marked female predominance [1]. Although most IHs involute without intervention, up to one quarter develop complications such as ulceration, bleeding, functional impairment, or permanent disfigurement, with ulceration being the most frequent and clinically significant [1, 2, 3]. In contrast, vascular malformations are increasingly recognized as genetically driven mosaic disorders caused by somatic activating mutations in genes regulating endothelial growth and survival, including *PIK3CA*, *TEK/TIE2*, *AKT1*, *KRAS*, and *NRAS*. These variants result in constitutive activation of the PI3K/AKT/mTOR and RAS/MAPK pathways, leading to abnormal vascular morphogenesis, progressive lesion expansion, and chronic morbidity [4, 5]. This molecular insight has fundamentally shifted the conceptual framework of vascular anomalies from static structural defects toward dynamic, pathway‐dependent diseases.

Historically, treatment of VTMs relied on corticosteroids, surgery, laser therapy, embolization, or sclerotherapy, approaches limited by incomplete responses, recurrence, and procedure‐related morbidity. A major paradigm shift occurred in 2008 with the discovery that propranolol induces rapid regression of proliferating IHs, establishing β‐blockade as first‐line systemic therapy and the first widely adopted mechanism‐based pharmacologic intervention in the field [6, 7]. Subsequent studies demonstrated that propranolol modulates angiogenic signalling, endothelial survival pathways, vascular permeability, and the lesion microenvironment. In parallel, advances in molecular genetics enabled the development and repurposing of targeted agents, including inhibitors of PI3K/AKT/mTOR and RAS/MAPK signalling. Agents such as sirolimus, alpelisib, miransertib, and MEK inhibitors have shown clinically meaningful activity in complex or refractory disease, supporting a precision‐medicine approach to vascular anomalies. Together, these advances underscore a transition from empiric management toward rational, pathway‐directed therapy and position vascular anomalies as a human model for studying endothelial pathway addiction, somatic mosaicism, and therapeutic plasticity. Collectively, these discoveries position vascular anomalies as a model disease of endothelial signalling dysregulation, in which aberrant pathway activation, cellular plasticity, and microenvironmental cues converge to drive pathological vascular growth. Understanding how endothelial cells integrate PI3K/AKT/mTOR and RAS/MAPK signalling provides a unique framework linking developmental biology with therapeutic vulnerability. This review therefore focuses on vascular anomalies as a paradigm of pathway‐dependent endothelial disease and translational cellular medicine.

## 2 Methods

This narrative review was conducted using a structured literature search strategy. PubMed, Embase, and ClinicalTrials.gov were searched for articles published up to December 2025. Search terms included combinations of infantile hemangiomas, vascular malformations, PIK3CA, AKT, mTOR, RAS/MAPK, beta‐blockers, sirolimus, alpelisib, and targeted therapy. We included clinical trials, observational studies, case series, and key mechanistic studies relevant to endothelial biology and vascular development. Particular emphasis was placed on studies providing translational insight linking molecular pathways to therapeutic response. Reference lists of relevant reviews were screened manually to identify additional pertinent publications. Non‐English articles without available translations were excluded.

## 3 β‐Blockers in Infantile Hemangiomas

**FIGURE 1:**
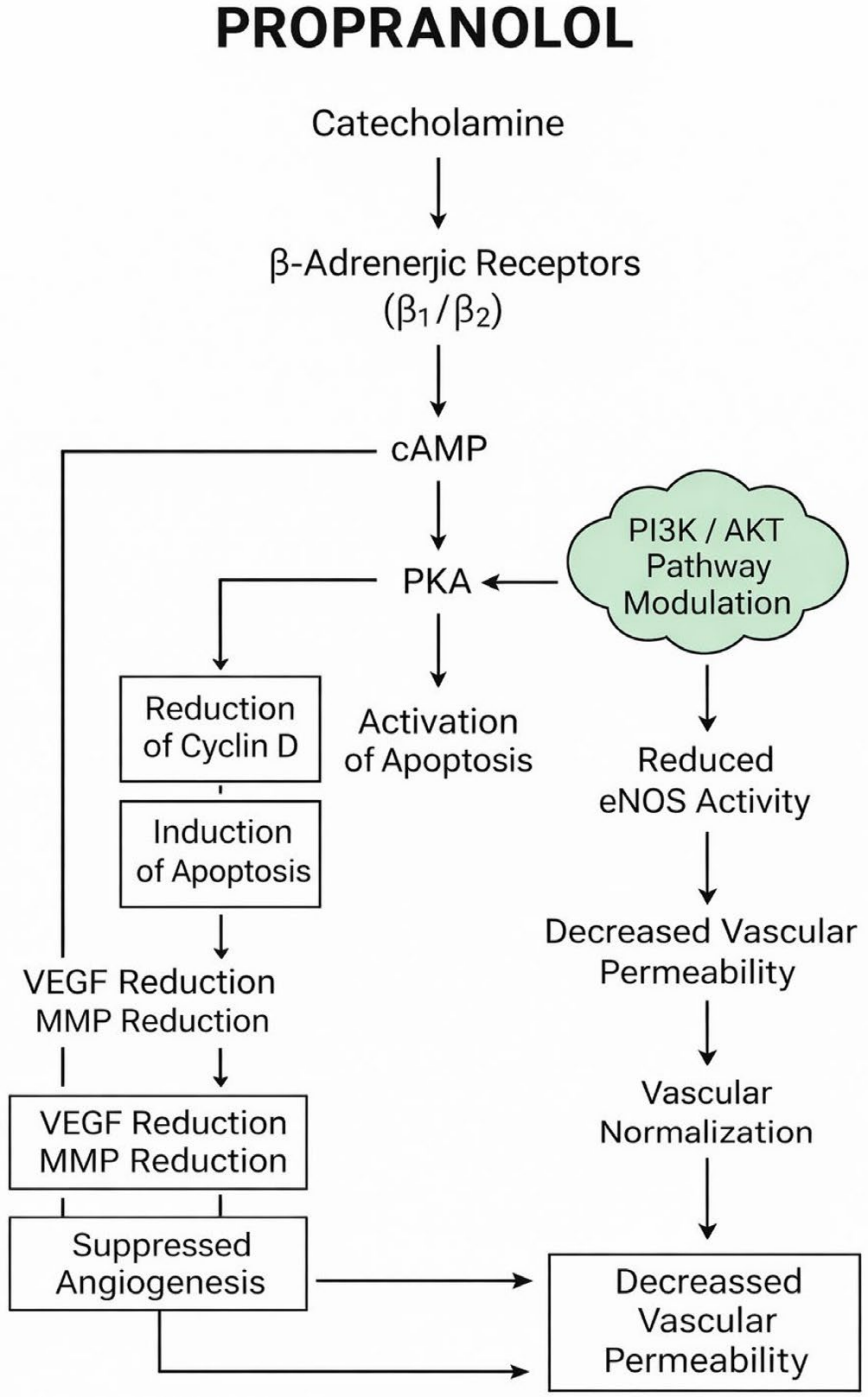
Molecular and vascular mechanisms underlying the therapeutic effects of propranolol. Propranolol, a non‐selective β‐adrenergic receptor antagonist, blocks catecholamine‐driven activation of β₁‐ and β₂‐adrenergic receptors, resulting in attenuation of the cAMP–protein kinase A (PKA) signalling axis. Inhibition of PKA activity leads to downregulation of cyclin D expression and promotes apoptosis, thereby suppressing cellular proliferation. Concurrently, propranolol modulates the PI3K/AKT signalling pathway, reducing endothelial nitric oxide synthase (eNOS) activity and subsequently decreasing vascular permeability. These effects contribute to vascular normalization and stabilization of the endothelial barrier. In addition, propranolol downregulates pro‐angiogenic mediators, including vascular endothelial growth factor (VEGF) and matrix metalloproteinases (MMPs), leading to inhibition of angiogenesis. Collectively, these interconnected molecular and vascular effects account for the anti‐proliferative, pro‐apoptotic, anti‐angiogenic, and vessel‐normalizing properties of propranolol.

Since the initial report in 2008, oral propranolol has become the first‐line systemic therapy for problematic proliferating infantile hemangiomas (IHs), while topical timolol is widely used for thin superficial lesions [6, 8, 9, 10]. In a pivotal randomized, placebo‐controlled trial, propranolol demonstrated superior clearance compared with placebo and established a standard regimen of 3 mg/kg/day for 6 months in infants requiring systemic therapy [7]. Beyond early vasoconstriction, β‐blockade exerts pleiotropic, lesion‐specific effects, including inhibition of β‐adrenergic signalling with reduced cAMP/PKA activity and downregulation of pro‐angiogenic programs such as VEGF and matrix metalloproteinases, thereby limiting endothelial migration and capillary morphogenesis [11, 12, 13, 14, 15, 16]. Convergent modulation of endothelial survival pathways, including PI3K/AKT/eNOS signalling, promotes involution through reduced proliferative drive and induction of apoptosis and autophagy in hemangioma endothelium [14, 16, 17, 18, 19, 20, 21, 22, 23, 24]. Propranolol additionally decreases vascular permeability and edema, consistent with vascular normalization, and exhibits β‐receptor–independent activity, including ANGPTL4 downregulation [25, 26, 27]. Although propranolol is classically described as a non‐selective β‐adrenergic receptor antagonist, direct experimental evidence demonstrating that its therapeutic efficacy in infantile hemangioma is mediated primarily through β‐adrenergic receptor blockade remains limited. Early mechanistic studies suggesting β‐independent activity relied largely on murine endothelioma models, such as bEnd.3 cells, which do not fully recapitulate the biology of human infantile hemangioma [28]. More recently, disease‐relevant experimental studies using primary human infantile hemangioma endothelial cells isolated from patient lesions have provided compelling evidence for a β‐adrenergic receptor–independent mechanism of propranolol action. SOX18, a transcription factor critical for vascular and lymphatic endothelial specification, has emerged as a central regulatory node in infantile hemangioma (IH) biology. Experimental work by Overman et al., published in eLife (2019), identified R(+)‐propranolol as a small‐molecule inhibitor of SOX18 transcriptional activity, demonstrating suppression of endothelial differentiation programs and vasculogenic potential [29]. These findings were extended by Seebauer et al. in the Journal of Clinical Investigation (2022), who demonstrated that non‐β‐blocking enantiomers of propranolol and atenolol inhibit vasculogenesis in IH‐derived endothelial cells independently of canonical β‐adrenergic receptor signalling [30]. The study provided mechanistic evidence that SOX18 inhibition disrupts downstream developmental and angiogenic pathways central to hemangioma biology. The principal molecular pathways involved in propranolol‐mediated modulation of endothelial proliferation, apoptosis, and angiogenesis are illustrated in Figure 1.

Collectively, these data reframe propranolol not merely as a hemodynamic modulator but as a transcriptional regulator influencing endothelial lineage specification and vascular development. In light of these findings, propranolol therapy for infantile hemangioma should not be viewed as a fully elucidated mechanism‐based or pathway‐directed pharmacologic intervention. Rather, it represents an effective empiric therapy with emerging, but still evolving, mechanistic understanding. Accordingly, references to propranolol as a mechanism‐based or pathway‐directed therapy have been revised to more accurately reflect the current state of experimental evidence. Clinically, earlier initiation during the proliferative phase is associated with improved outcomes and fewer residual sequelae, whereas delayed treatment increases the likelihood of incomplete response [31, 32, 33]. Segmental IHs and lesions in high‐risk anatomic sites more frequently require systemic therapy due to ulceration, functional compromise, or disfigurement risk [34, 35]. Objective monitoring may assist in treatment duration and tapering and reduce recurrence after discontinuation [36]. Propranolol is typically initiated at 1 mg/kg/day and titrated to 2–3 mg/kg/day, with monitoring for cardiovascular and respiratory adverse effects and caregiver education to prevent hypoglycemia, which remains rare but clinically significant, particularly during fasting or intercurrent illness [7, 10, 37]. Supportive wound care is essential for ulcerated IHs, with individualized dosing to balance ulcer healing and lesion regression [38]. Overall, propranolol remains the reference systemic agent for IHs, with topical timolol preferred for small superficial lesions. Topical timolol provides modest but clinically meaningful improvement, particularly when initiated early, with minimal systemic exposure [39, 40, 41, 42]. In infants with contraindications or intolerance to propranolol, β1‐selective atenolol and long‐acting nadolol demonstrate comparable efficacy with potential reductions in selected adverse effects [43, 44, 45, 46, 47, 48]. In contrast, most vascular malformations are genetically defined mosaic disorders that rarely respond to adrenergic blockade, reinforcing the shift toward molecular diagnostics and pathway‐directed therapies targeting PI3K/AKT/mTOR and RAS/MAPK signalling [49].

## 4 Molecular Targeted Therapies: PI3K/AKT/mTOR Pathway Inhibition

**FIGURE 2:**
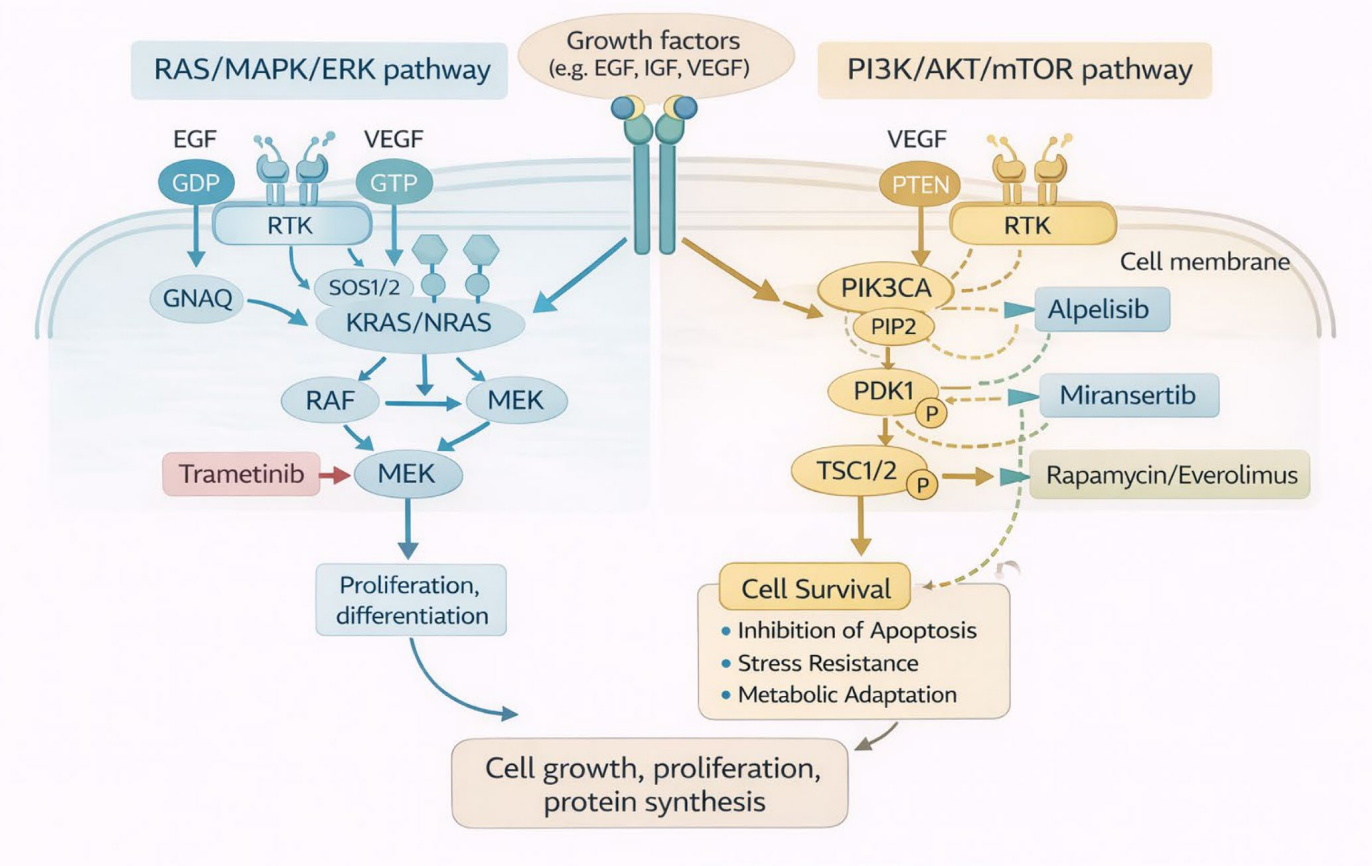
Schematic representation of the RAS/MAPK/ERK and PI3K/AKT/mTOR signalling pathways. The figure illustrates two key intracellular signalling pathways activated downstream of receptor tyrosine kinases (RTKs) upon stimulation with growth factors such as EGF, IGF, and VEGF. On the left, the RAS/MAPK/ERK pathway, associated with fast‐flow vascular anomalies, signals via GNAQ, KRAS/NRAS, RAF, and MEK, leading to cellular proliferation and differentiation. This cascade is inhibited by Trametinib, a selective MEK inhibitor. On the right, the PI3K/AKT/mTOR pathway, characteristic of slow‐flow vascular malformations, is activated through PIK3CA, PDK1, AKT, TSC1/2, and mTORC1, promoting cell survival, growth, and protein synthesis, with downstream effects including inhibition of apoptosis, enhanced stress resistance, and metabolic adaptation. This pathway is negatively regulated by PTEN and can be pharmacologically targeted at multiple nodes: Alpelisib (PI3Kα inhibitor), Miransertib (AKT inhibitor), and Rapamycin/Everolimus (mTOR inhibitors). The convergence of both pathways on shared outputs such as cell growth and protein synthesis highlights their complementary roles in regulating endothelial function and pathological signalling in vascular anomalies.

Recent advances in molecular genetics have identified somatic activating mutations in the *PIK3CA* gene as major drivers of a broad spectrum of vascular malformations, particularly venous malformations (VMs), lymphatic malformations (LMs), and combined lesions. These mutations result in constitutive activation of the PI3K/AKT/mTOR signalling pathway, a central regulator of endothelial cell growth, proliferation, metabolism, and angiogenesis. Aberrant activation of this pathway promotes uncontrolled endothelial expansion, dysregulated vascular morphogenesis, increased vascular permeability, and progressive tissue overgrowth, thereby underpinning the clinical phenotype of *PIK3CA*‐related vascular anomalies and overgrowth syndromes [4]. Recognition of this molecular basis has marked a paradigm shift from empiric symptom management toward precision‐targeted therapy. The major signalling pathways involved in vascular anomalies, including PI3K/AKT/mTOR and RAS/MAPK cascades, are schematically illustrated in Figure 2.

### 4.1 mTOR Inhibition: Sirolimus and Everolimus

Sirolimus (rapamycin), an oral mTORC1 inhibitor, has emerged as a cornerstone therapy for complex and refractory vascular malformations driven by chronic PI3K/AKT/mTOR pathway activation. In vascular anomalies, mTOR hyperactivation promotes excessive endothelial proliferation, aberrant angiogenesis, and increased vascular permeability. mTORC1 inhibition by sirolimus suppresses endothelial growth and pathological vessel formation, thereby stabilizing lesion progression. Mechanistically, sirolimus inhibits HIF‐1α–dependent VEGF expression, attenuating angiogenic signalling under hypoxic conditions [50, 51, 52], and interferes with cell‐cycle regulators including cyclin D1 and p70S6 kinase, resulting in reduced endothelial proliferation [53]. In parallel, sirolimus decreases eNOS activity and nitric oxide production, improving vascular permeability and edema characteristic of venous and lymphatic malformations [54], and promotes vascular maturation through enhanced pericyte–endothelial interactions, leading to more stable vessels [55]. Reduced circulating endothelial progenitor cells and impaired lesion recruitment further contribute to disease modification. Clinical efficacy of sirolimus has been demonstrated in prospective trials and real‐world cohorts. Significant improvements in lesion size, pain, bleeding, and functional impairment were reported in patients with complicated vascular anomalies refractory to conventional therapies, with manageable toxicity dominated by hematologic adverse events [56]. The Phase III VASE trial confirmed these findings in paediatric and adult patients with slow‐flow malformations, demonstrating clinical improvement in approximately 85% of patients within 12 months, frequently within the first month of therapy [57]. However, symptom recurrence after treatment discontinuation occurred in more than half of patients, indicating a need for prolonged or maintenance therapy. Meta‐analytic data from prospective studies further support consistent lesion volume reduction and functional improvement with predominantly mild and reversible adverse effects [57]. Paediatric cohorts, particularly in head and neck lymphatic malformations, show response rates exceeding 90%, with improvements in pain, swelling, airway compromise, and cosmetic outcomes [58, 59]. The PERFORMUS trial demonstrated clinically meaningful symptom improvement even in the absence of marked volumetric MRI changes [60]. Real‐world pharmacokinetic data suggest that lower sirolimus trough levels (4–10 ng/mL) may be sufficient for efficacy, especially in children, who often exhibit faster responses and fewer adverse events than adults [61]. Long‐term therapy nevertheless requires careful monitoring for immunosuppression, metabolic disturbances, impaired wound healing, and infection risk. Although sirolimus and everolimus are sometimes discussed as emerging or experimental therapies for infantile hemangioma, their use is supported by both experimental and clinical evidence. Preclinical studies published in 2011 and 2014 demonstrated that rapamycin modulates hemangioma endothelial cell proliferation and differentiation, providing a mechanistic rationale for targeting the PI3K/AKT/mTOR pathway in this disease [62, 63]. Subsequently, accumulating evidence from clinical reports, case series, and systematic analyses has supported the use of sirolimus in the management of infantile hemangioma, particularly in cases refractory to first‐line therapies or associated with significant complications. Early multicentre experience with topical sirolimus demonstrated its potential efficacy and favourable safety profile in superficial vascular anomalies, including infantile hemangiomas, as reported by Dodds et al. in Paediatric Dermatology [64]. Further clinical data from a single‐centre paediatric cohort published in the Hong Kong Journal of Paediatrics [65] confirmed the therapeutic benefit of systemic sirolimus in benign vascular anomalies resistant to conventional treatment. More recently, Dekeuleneer et al. [66] described successful short‐term sirolimus therapy in a case of propranolol‐resistant infantile hemangioma, highlighting its role as a rescue treatment option. These findings are reinforced by a recent systematic review by Abtahi‐Naeini et al. [67, 68], which concluded that sirolimus appears to be an effective and generally well‐tolerated therapeutic alternative for selected cases of infantile hemangioma, although larger prospective studies are still needed to define optimal dosing strategies and long‐term safety. Collectively, these studies indicate that mTOR inhibition in infantile hemangioma is not merely hypothetical but is grounded in both translational and clinical experience. Everolimus, a structurally related mTOR inhibitor, has shown activity in selected vascular tumours and malformations. Case reports describe successful use in neonates with giant hepatic hemangiomas and Kasabach–Merritt syndrome refractory to standard therapy, as well as sustained benefit in epithelioid hemangiomas resistant to sclerotherapy [68, 69, 70]. Preclinical studies demonstrate potent inhibition of hemangioma‐derived endothelial proliferation and induction of p53‐mediated apoptosis, with stronger antiproliferative effects than propranolol in vitro [70]. Although controlled clinical trials are lacking, these data support everolimus as a potential alternative in refractory cases.

### 4.2 PI3Kα Inhibition: Alpelisib

Alpelisib, a selective PI3Kα inhibitor, represents a major advance in the treatment of PIK3CA‐driven vascular anomalies by directly targeting the mutant catalytic subunit responsible for pathway hyperactivation. Inhibition of PI3Kα suppresses downstream AKT and mTOR signalling, reducing pathological endothelial proliferation and vascular overgrowth. Early clinical experience demonstrates significant reductions in lesion volume, improvement in pain and function, and amelioration of venous overload–related complications, with a manageable safety profile dominated by hyperglycemia and gastrointestinal adverse events [71]. The translational relevance of PI3Kα inhibition was established by Venot et al., who showed that alpelisib (BYL719) reversed disease phenotypes in PIK3CA‐related overgrowth models and produced consistent clinical benefit in patients with PROS, including CLOVES syndrome [72]. These findings were extended to capillary–venous malformations driven by PIK3CA or TEK mutations, where alpelisib induced regression of established lesions and improved survival in preclinical models [73].

### 4.3 AKT Inhibition and Pathway Resistance

AKT inhibition has emerged as an additional therapeutic strategy for PI3K/AKT/mTOR–driven vascular anomalies, particularly those caused by activating AKT1 variants such as Proteus syndrome. Preclinical studies demonstrate that AKT signalling is required for both initiation and maintenance of vascular malformations, and pharmacologic inhibition with agents such as miransertib induces regression of established lesions [74]. Clinically, miransertib has shown benefit in mosaic overgrowth disorders, improving vascular features and slowing disease progression in patients refractory to mTOR inhibition [75, 76, 77]. Other AKT inhibitors, including MK‐2206, remain under investigation, although clinical experience in vascular anomalies is limited [78]. Despite these advances, therapeutic challenges persist. Molecular heterogeneity among vascular malformations leads to variable responses depending on the underlying genetic alteration and degree of pathway activation. Adverse effects, including immunosuppression, hyperlipidemia, hyperglycemia, and infection risk, constrain dose escalation and long‐term tolerability. In addition, resistance mechanisms—frequently involving compensatory activation of parallel pathways such as RAS/RAF/MEK/ERK or sustained mTORC2 signalling—highlight the need for combination strategies and precise molecular stratification [79].

## 5 Anti‐VEGF Therapies and Novel Therapies

### 5.1 Anti‐VEGF Therapies

Vascular endothelial growth factor (VEGF) is a key regulator of angiogenesis, driving endothelial proliferation, migration, and neovessel formation, and dysregulated VEGF signalling has been implicated in infantile hemangiomas and vascular malformations, including cavernous malformations and arteriovenous malformations. Accordingly, VEGF inhibition has been explored as a therapeutic strategy. Bevacizumab, a monoclonal antibody targeting VEGF‐A, demonstrated anti‐angiogenic efficacy in preclinical models, including attenuation of VEGF‐driven vascular malformations in adult mouse brain models [80]. Clinical evidence remains limited and heterogeneous, with case reports and small series suggesting reduced lesion size, vascular permeability, and symptom burden, primarily in multimodal treatment settings. In early proliferating infantile hemangiomas, intralesional bevacizumab showed initial efficacy comparable to intralesional triamcinolone acetonide, but corticosteroid therapy achieved superior outcomes with repeated treatment, underscoring the limited efficacy of isolated VEGF blockade [80]. Although anti‐VEGF‐A strategies have demonstrated anti‐angiogenic activity in preclinical models, their clinical efficacy in infantile hemangioma appears limited when compared with corticosteroid therapy. Importantly, a pivotal study published in the New England Journal of Medicine in 2010 demonstrated that systemic corticosteroids profoundly suppress VEGF‐A expression in infantile hemangioma endothelial cells at the transcriptional level [81]. This mechanistic finding provides a biological explanation for the superior clinical efficacy of corticosteroids relative to isolated VEGF‐A inhibition and underscores why anti‐VEGF therapy alone may be insufficient in this context. Overall, current data support an adjunctive rather than primary role for anti‐VEGF therapy in vascular anomalies, given modest efficacy and concerns regarding wound healing, bleeding risk, and systemic toxicity.

### 5.2 Pan‐PI3K Inhibitors and Broad‐Spectrum Targeting

Recognition of molecular heterogeneity in vascular malformations has stimulated interest in broad‐spectrum inhibitors capable of targeting multiple PI3K isoforms simultaneously. KTC1101, a pan‐class I PI3K inhibitor, has shown promising preclinical activity by suppressing vascular lesion growth and modulating immune responses. In oncologic models, KTC1101 demonstrated synergistic activity with immune checkpoint inhibitors, particularly anti–PD‐1 therapy, suggesting potential applicability in complex vascular anomalies characterized by multifocal pathway activation [82]. Although clinical data in vascular malformations are currently lacking, such agents may represent future therapeutic options for lesions resistant to isoform‐specific inhibition. Buparlisib (BKM120), another pan‐PI3K inhibitor, has been evaluated in oncologic trials, including combination regimens with bevacizumab in metastatic renal cell carcinoma, where safety and efficacy signals were observed [83]. Its ability to inhibit all PI3K isoforms provides a theoretical advantage in malformations driven by heterogeneous or overlapping molecular alterations. However, neuropsychiatric and metabolic adverse effects have limited its broader clinical adoption, and experience in vascular anomalies remains investigational.

## 6 RAS/MAPK Pathway Inhibition

Parallel to PI3K pathway dysregulation, aberrant activation of the RAS/MAPK signalling cascade has emerged as a key driver of fast‐flow vascular anomalies, particularly AVMs and complex lymphatic flow disorders. Somatic activating mutations in *KRAS* and *NRAS* have been increasingly identified in sporadic AVMs and syndromic vascular lesions. In endothelial cell–specific In an endothelial‐specific KRAS‐driven mouse model, Nguyen et al. demonstrated in *Human Molecular Genetics* (2023) that sustained MAPK activation is required not only for lesion initiation but also for maintenance, and that pharmacologic MEK inhibition with trametinib reversed vascular dysplasia and improved survival [84]. These findings provided strong experimental evidence for pathway addiction in RAS‐driven vascular lesions [85]. Clinical translation of MEK inhibition has yielded encouraging results in patients with refractory vascular anomalies. Case series and early clinical reports describe significant improvement in AVMs unresponsive to surgery or embolization, as well as in life‐threatening lymphatic anomalies such as kaposiform lymphangiomatosis associated with *NRAS* mutations [86]. Improvements in coagulopathy, pulmonary function, and overall clinical status have been observed. Additionally, MEK inhibitors have demonstrated efficacy in lymphatic flow disorders linked to germline RAS pathway variants, including Noonan syndrome [87]. Although current evidence is limited to small cohorts and compassionate‐use experiences, these findings support MEK inhibition as a rational, pathway‐directed therapeutic strategy warranting further systematic evaluation.

### 6.1 Direct KRAS Inhibition

A major conceptual advance in vascular anomaly therapeutics has been the direct pharmacologic targeting of oncogenic *KRAS* variants, long considered “undruggable.” The development of covalent inhibitors targeting the *KRAS* G12C mutation has enabled unprecedented precision targeting. In a landmark translational study, Fraissenon et al. demonstrated that sotorasib, a *KRAS* G12C–specific inhibitor, significantly reduced lesion burden and improved survival in mouse models of mosaic *KRAS* G12C–driven AVMs [88]. Importantly, compassionate‐use administration of sotorasib in two adult patients with severe, treatment‐refractory AVMs harboring *KRAS* G12C mutations resulted in rapid clinical improvement and substantial radiologic regression without serious adverse events. These findings not only validate *KRAS* as a therapeutic target in vascular anomalies but also exemplify the extension of precision oncology paradigms to non‐neoplastic vascular disorders.

## 7 Repurposed and Emerging Therapies

Beyond pathway‐specific inhibitors, drug repurposing has emerged as an attractive strategy to expand therapeutic options for vascular tumours and malformations. Itraconazole, a widely used antifungal agent, has gained attention for its anti‐angiogenic properties mediated through inhibition of the Hedgehog (HH) signalling pathway. Preclinical studies in hemangioma‐derived endothelial cells demonstrated reduced proliferation, suppressed VEGF expression, and induction of apoptosis following itraconazole exposure, effects that were reversed by HH pathway activation [89]. Clinically, itraconazole has shown encouraging activity in small prospective studies and case reports, including superior response rates compared with propranolol in a limited paediatric cohort and successful treatment of combined cutaneous and hepatic hemangiomas without significant toxicity [90, 91]. While promising, these findings require validation in larger, controlled trials before routine clinical adoption. In parallel, computational and transcriptomic approaches have identified additional candidate therapeutics. Weighted gene co‐expression network analysis of infantile hemangioma datasets revealed enrichment of PI3K/AKT/mTOR, RAS/MAPK, and cGMP/PKG pathways, enabling in silico identification of repurposable drugs with predicted anti‐proliferative effects. Subsequent in vitro validation confirmed that several agents, including histone deacetylase inhibitors, and tyrosine kinase inhibitors effectively suppressed proliferation and migration of hemangioma‐derived endothelial cells while promoting apoptosis [92]. These data highlight the potential of systems biology–driven drug discovery to accelerate therapeutic innovation in vascular anomalies.

## 8 Genomic Profiling and Precision Diagnostics

Advances in genomic sequencing have further transformed the therapeutic landscape of vascular anomalies. Ultra‐deep sequencing techniques now enable detection of pathogenic somatic variants at very low variant allele fractions, even in mosaic or surgically inaccessible lesions. In a large cohort study, molecular diagnoses were achieved in a substantial proportion of patients with complex lymphatic and vascular malformations, and genotype‐guided therapy resulted in measurable clinical improvement in the majority of treated individuals [93]. Importantly, minimally invasive approaches such as liquid biopsy of plasma or lymphatic fluid have expanded access to molecular diagnostics, facilitating precision therapy selection while reducing procedural risk.

## 9 Future Perspectives

Together, these emerging therapeutic strategies—ranging from anti‐VEGF antibodies and pan‐PI3K inhibitors to MEK inhibitors, direct *KRAS* targeting, and repurposed agents—illustrate the rapid evolution of mechanism‐based treatment for vascular anomalies. While many approaches remain investigational, they underscore a shift toward individualized therapy guided by molecular profiling. Continued prospective trials, long‐term safety monitoring, and integration of genomic diagnostics will be essential to define optimal sequencing, combination strategies, and durability of response. Ultimately, these advances promise to further refine precision‐medicine frameworks for patients with complex and refractory vascular anomalies.

## 10 Proposed Therapeutic Decision Framework for Infantile Hemangiomas and Vascular Malformations

Therapeutic decision‐making should integrate clinical phenotype, flow characteristics, and molecular drivers. In infantile hemangiomas, first‐line therapy remains non‐selective β‐blockers, reflecting their impact on vasoconstriction, angiogenic signalling, and endothelial metabolism. In cases of refractoriness or intolerance, evaluation of downstream signalling activation, including PI3K/AKT/mTOR, may support consideration of targeted agents in selected scenarios. For vascular malformations, initial stratification according to flow dynamics (slow‐flow vs. fast‐flow) and dominant tissue involvement is critical. In extensive or progressive slow‐flow malformations, molecular testing for PIK3CA, TEK, or AKT1 variants provides a rational basis for mTOR or PI3K pathway inhibition. Conversely, fast‐flow malformations or lesions associated with RAS/MAPK activation may benefit from pathway‐directed approaches targeting MEK or related nodes, ideally within clinical trials. Across entities, treatment selection should consider expected durability of response, potential pathway escape mechanisms, and long‐term safety, emphasizing the need for biomarker‐guided therapy and longitudinal monitoring.

## 11 Conclusion

Over the past two decades, vascular anomaly care has shifted from largely procedural symptom control toward mechanism‐informed pharmacotherapy. Beta‐blockers, enabled by the discovery of propranolol efficacy in infantile hemangiomas, provided the first standardized systemic treatment in this field and demonstrated that targeting lesion‐specific biology can alter natural history and reduce functional and cosmetic morbidity. More recently, recurrent somatic variants affecting PI3K/AKT/mTOR and RAS/MAPK signalling have reframed many vascular malformations as mosaic, genetically defined disorders. This has enabled rational use of targeted agents such as sirolimus and alpelisib, with additional options emerging for AKT‐, MEK‐ and mutation‐specific inhibition in selected contexts. Next steps for the field include broader access to sensitive molecular diagnostics (including low‐allele‐fraction testing and fluid‐based approaches), prospective genotype‐stratified trials, and long‐term safety and durability monitoring. Vascular anomalies may also serve as a model for precision medicine beyond oncology, where somatic mosaicism, pathway addiction and targeted therapy converge to refine disease classification and individualized treatment. An overview of currently used and emerging pharmacologic therapies for vascular tumours and malformations is summarized in Table 1.

**TABLE 1:** Overview of pharmacologic targeted therapies currently used or under investigation in vascular tumours and malformations. Therapeutic classes are summarized according to molecular targets, predominant lesion types, clinical efficacy, safety considerations, and stage of clinical adoption. IHs—infantile hemangiomas; VMs—venous malformations; LMs—lymphatic malformations; PROS—PIK3CA‐related overgrowth spectrum.

Therapeutic class	Representative agents	Primary molecular target/pathway	Predominant lesion types	Clinical efficacy	Safety profile	Clinical status/remarks
β‐Blockers	Propranolol, Timolol, Atenolol, Nadolol	β‐adrenergic receptors; downstream inhibition of VEGF, cAMP/PKA, PI3K/AKT/eNOS signalling	Infantile hemangiomas (IHs)	High efficacy in proliferating IHs; rapid regression and long‐term control	Generally well tolerated; risk of hypoglycemia, bradycardia, hypotension, bronchospasm (systemic therapy)	Established first‐line therapy for IHs; topical agents preferred for superficial lesions
mTOR inhibitors	Sirolimus, Everolimus	mTORC1 inhibition within PI3K/AKT/mTOR pathway	Venous malformations (VMs), lymphatic malformations (LMs), combined slow‐flow lesions	High response rates in refractory and extensive lesions; symptom and volume reduction	Immunosuppression, mucositis, hyperlipidemia, cytopenias; requires laboratory monitoring	Established targeted therapy for complex slow‐flow malformations; often long‐term treatment
PI3Kα inhibitors	Alpelisib	Selective PI3Kα inhibition upstream of AKT/mTOR	*PIK3CA*‐related vascular malformations and PROS	Promising lesion regression and symptom improvement in genetically confirmed cases	Hyperglycemia, diarrhoea, rash; metabolic monitoring required	Emerging precision therapy; compassionate use and early clinical trials
AKT inhibitors	Miransertib	AKT inhibition within PI3K/AKT/mTOR axis	*AKT1*‐driven malformations (e.g., Proteus syndrome), refractory PROS	Clinical and radiologic improvement in selected cases	Gastrointestinal symptoms, metabolic effects; limited long‐term data	Investigational; option for mTOR/PI3K‐resistant disease
MEK inhibitors	Trametinib	MEK inhibition within RAS/MAPK pathway	Arteriovenous malformations (AVMs), RAS‐driven lymphatic anomalies	Promising responses in refractory AVMs and lymphatic disorders	Dermatologic toxicity, cardiomyopathy risk; close monitoring required	Investigational/compassionate use
Anti‐VEGF agents	Bevacizumab	VEGF ligand neutralization	Selected IHs, cavernous malformations, AVMs	Variable efficacy; often adjunctive	Hypertension, impaired wound healing, bleeding risk	Adjunctive/limited role
Repurposed agents	Itraconazole, Thalidomide	Hedgehog pathway inhibition; anti‐angiogenic effects	Infantile hemangiomas, selected malformations	Preliminary efficacy in small studies and case reports	Drug‐specific adverse effects; limited paediatric data	Experimental/off‐label

## Author Contributions

**Hubert Arasiewicz:** conceptualization, investigation, writing – original draft, methodology, validation, visualization, writing – review and editing, project administration, formal analysis, software, data curation, supervision, resources, funding acquisition.

## Funding

The authors have nothing to report.

## Conflicts of Interest

The authors declare no conflicts of interest.

## Data Availability

Data sharing not applicable to this article as no datasets were generated or analysed during the current study.

## References

[1] Y. Sun , J. Zhao , Y. Meng , et al., “The Prevalence, Complications, and Risk Factors for Infantile Hemangioma: A Systematic Review and Meta‐Analysis,” International Journal of Dermatology 63, no. 6 (2024): 737–746, 10.1111/ijd.17062. 38329175

[2] A. Rešić , Z. Barčot , D. Habek , Z. Pogorelić , and M. Bašković , “The Evaluation, Diagnosis, and Management of Infantile Hemangiomas—A Comprehensive Review,” Journal of Clinical Medicine 14 (2025): 425, 10.3390/jcm14020425. 39860430 PMC11765582

[3] E. Fernández Faith , S. D. Shah , M. Braun , et al., “Incidence and Clinical Factors Associated With Ulceration in Infantile Hemangiomas,” Journal of the American Academy of Dermatology 88, no. 2 (2023): 414–420, 10.1016/j.jaad.2022.10.047. 36404484

[4] S. D. Castillo , E. Baselga , and M. Graupera , “PIK3CA Mutations in Vascular Malformations,” Current Opinion in Hematology 26, no. 3 (2019): 170–178, 10.1097/MOH.0000000000000496. 30855339

[5] N. L. Busaidy , P. LoRusso , K. Lawhorn , et al., “The Prevalence and Impact of Hyperglycemia and Hyperlipidemia in Patients With Advanced Cancer Receiving Combination Treatment With the mTOR Inhibitor Temsirolimus and IGF‐1R Antibody Cixutumumab,” Oncologist 20, no. 7 (2015): 737–741, 10.1634/theoncologist.2015-0065. 26054632 PMC4492245

[6] C. Léauté‐Labrèze , E. Dumas de la Roque , T. Hubiche , F. Boralevi , J. B. Thambo , and A. Taïeb , “Propranolol for Severe Hemangiomas of Infancy,” New England Journal of Medicine 358 (2008): 2649–2651, 10.1056/NEJMc0708819. 18550886

[7] C. Léauté‐Labrèze , P. Hoeger , J. Mazereeuw‐Hautier , et al., “A Randomized, Controlled Trial of Oral Propranolol in Infantile Hemangioma,” New England Journal of Medicine 372, no. 8 (2015): 735–746, 10.1056/NEJMoa1404710. 25693013

[8] C. B. Bayart and H. A. Brandling‐Bennett , “Beta‐Blockers for Childhood Vascular Tumors,” Current Opinion in Pediatrics 27, no. 4 (2015): 454–459, 10.1097/MOP.0000000000000238. 26087423

[9] J. C. Lee , O. Modiri , R. W. England , C. J. Shawber , and J. K. Wu , “Propranolol Therapy in Infantile Hemangioma: It Is Not Just About the Beta,” Plastic and Reconstructive Surgery 147, no. 4 (2021): 875–885, 10.1097/PRS.0000000000007699. 33776033

[10] X. Tan , S. Guo , and C. Wang , “Propranolol in the Treatment of Infantile Hemangiomas,” Clinical, Cosmetic and Investigational Dermatology 14 (2021): 1155–1163, 10.2147/CCID.S332625. 34511960 PMC8423716

[11] W. Parichatikanond , R. Duangrat , H. Kurose , and S. Mangmool , “Regulation of β‐Adrenergic Receptors in the Heart: A Review on Emerging Therapeutic Strategies for Heart Failure,” Cells 13, no. 20 (2024): 1674, 10.3390/cells13201674. 39451192 PMC11506672

[12] X. Xu , J. Kaindl , M. J. Clark , et al., “Binding Pathway Determines Norepinephrine Selectivity for the Human β1AR Over β2AR,” Cell Research 31, no. 5 (2021): 569–579, 10.1038/s41422-020-00424-2. 33093660 PMC8089101

[13] J. Stiles , C. Amaya , R. Pham , et al., “Propranolol Treatment of Infantile Hemangioma Endothelial Cells: A Molecular Analysis,” Experimental and Therapeutic Medicine 4, no. 4 (2012): 594–604, 10.3892/etm.2012.654. 23170111 PMC3501380

[14] W. K. Pan , P. Li , Z. T. Guo , Q. Huang , and Y. Gao , “Propranolol Induces Regression of Hemangioma Cells via the Down‐Regulation of the PI3K/Akt/eNOS/VEGF Pathway,” Pediatric Blood & Cancer 62, no. 8 (2015): 1414–1420, 10.1002/pbc.25453. 25728347

[15] L. Zhang , H. M. Mai , J. Zheng , et al., “Propranolol Inhibits Angiogenesis via Down‐Regulating the Expression of Vascular Endothelial Growth Factor in Hemangioma Derived Stem Cell,” International Journal of Clinical and Experimental Pathology 7, no. 1 (2013): 48–55. 24427325 PMC3885459

[16] X. Wang and R. A. Khalil , “Matrix Metalloproteinases, Vascular Remodeling, and Vascular Disease,” Advances in Pharmacology 81 (2018): 241–330, 10.1016/bs.apha.2017.08.002. 29310800 PMC5765875

[17] P. Kaulanjan‐Checkmodine , S. Oucherif , S. Prey , et al., “Is infantile hemangioma a neuroendocrine tumor?,” International Journal of Molecular Sciences 23, no. 9 (2022): 5140, 10.3390/ijms23095140. 35563552 PMC9104933

[18] F. Moisan , S. Oucherif , P. Kaulanjan‐Checkmodine , et al., “Critical Role of Aquaporin‐1 and Telocytes in Infantile Hemangioma Response to Propranolol Beta Blockade,” Proceedings of the National Academy of Sciences of the United States of America 118, no. 7 (2021): e2018690118, 10.1073/pnas.2018690118. 33558238 PMC7896303

[19] Y. Ji , S. Chen , X. Xiao , S. Zheng , and K. Li , “β‐Blockers: A Novel Class of Antitumor Agents,” Oncotargets and Therapy 5 (2012): 391–401, 10.2147/OTT.S38403. 23226026 PMC3513911

[20] T. H. Yao , P. Pataer , K. P. Regmi , et al., “Propranolol Induces Hemangioma Endothelial Cell Apoptosis via a p53‐BAX Mediated Pathway,” Molecular Medicine Reports 18, no. 1 (2018): 684–694, 10.3892/mmr.2018.9013. 29767244 PMC6059697

[21] V. Albiñana , E. Gallardo‐Vara , J. Casado‐Vela , L. Recio‐Poveda , L. M. Botella , and A. M. Cuesta , “Propranolol: “pick and roll” team player in benign tumors and cancer therapies,” Journal of Clinical Medicine 11, no. 15 (2022): 4539, 10.3390/jcm11154539. 35956154 PMC9369479

[22] S. Qian , Z. Wei , W. Yang , J. Huang , Y. Yang , and J. Wang , “The Role of BCL‐2 Family Proteins in Regulating Apoptosis and Cancer Therapy,” Frontiers in Oncology 12 (2022): 985363, 10.3389/fonc.2022.985363. 36313628 PMC9597512

[23] B. Lorusso , G. Cerasoli , A. Falco , et al., “β‐Blockers Activate Autophagy on Infantile Hemangioma‐Derived Endothelial Cells in Vitro,” Vascular Pharmacology 146 (2022): 107110, 10.1016/j.vph.2022.107110. 36103993

[24] Y. Dai , F. Hou , L. Buckmiller , J. Bischoff , and F. Blei , “Decreased eNOS Protein Expression in Involuting and Propranolol‐Treated Hemangiomas,” Archives of Otolaryngology – Head & Neck Surgery 138, no. 2 (2012): 177–182, 10.1001/archoto.2011.1096. 22351865

[25] S. A. Patel , M. B. Nilsson , X. Le , T. Cascone , R. K. Jain , and J. V. Heymach , “Molecular Mechanisms and Future Implications of VEGF/VEGFR in Cancer Therapy,” Clinical Cancer Research 29, no. 1 (2023): 30–39, 10.1158/1078-0432.CCR-22-1366. 35969170 PMC10274152

[26] S. Xiang , X. Gong , T. Qiu , et al., “Insights Into the Mechanisms of Angiogenesis in Infantile Hemangioma,” Biomedicine & Pharmacotherapy 178 (2024): 117181, 10.1016/j.biopha.2024.117181. 39059349

[27] P. Ramos , Q. Shi , J. Kleberg , C. K. Maharjan , W. Zhang , and R. Kolb , “ANGPTL4: A Comprehensive Review of 25 Years of Research,” Cancers (Basel) 17, no. 14 (2025): 2364, 10.3390/cancers17142364. 40723247 PMC12293468

[28] M. Sasaki , P. E. North , J. Elsey , et al., “Propranolol Exhibits Activity Against Hemangiomas Independent of Beta Blockade,” npj Precision Oncology 3 (2019): 27, 10.1038/s41698-019-0099-9. 31701018 PMC6825155

[29] J. Overman , F. Fontaine , J. Wylie‐Sears , Y. Yang , T. M. Cox , and J. Bischoff , “R‐Propranolol Is a Small Molecule Inhibitor of the SOX18 Transcription Factor in a Rare Vascular Syndrome and Hemangioma,” eLife 8 (2019): e43026, 10.7554/eLife.43026. 31358114 PMC6667216

[30] C. T. Seebauer , M. S. Graus , L. Huang , et al., “Non‐Beta Blocker Enantiomers of Propranolol and Atenolol Inhibit Vasculogenesis in Infantile Hemangioma,” Journal of Clinical Investigation 132, no. 3 (2022): e151109, 10.1172/JCI151109. 34874911 PMC8803322

[31] R. J. Phillips , A. J. Penington , P. S. Bekhor , D. H. Penington , and J. B. Selva , “Use of Propranolol for Treatment of Infantile Haemangiomas in an Outpatient Setting,” Journal of Paediatrics and Child Health 48, no. 10 (2012): 902–906, 10.1111/j.1440-1754.2012.02552.x. 22897120

[32] J. M. C. Tan , H. W. Lim , and M. J.‐A. Koh , “Oral Propranolol for the Treatment of Infantile Hemangiomas in Singapore,” Singapore Medical Journal 62, no. 3 (2021): 139–142. 31989180 10.11622/smedj.2020008 PMC8027146

[33] A. Giachetti , M. S. Díaz , P. Boggio , and M. L. Posadas Martínez , “Early Propranolol Treatment of Infantile Hemangiomas Improves Outcome,” Anais Brasileiros de Dermatologia 98, no. 3 (2023): 310–315, 10.1016/j.abd.2022.04.008. 36577593 PMC10173064

[34] T. Qiu , K. Yang , S. Dai , S. Chen , and Y. Ji , “Clinical Features of Segmental Infantile Hemangioma: A Prospective Study,” Therapeutics and Clinical Risk Management 17 (2021): 119–125, 10.2147/TCRM.S291059. 33536759 PMC7850443

[35] S. Tiple , P. Kimmatkar , S. Das , et al., “Treatment Outcomes of Oral Propranolol in Periocular Infantile Capillary Hemangioma and Factors Predictive of Recurrence and Incomplete Resolution: A Multi‐Centric Study,” Oman J Ophthalmol 16, no. 1 (2023): 75–81, 10.4103/ojo.ojo_11_22. 37007245 PMC10062112

[36] Y. Wu , P. Zhao , W. Song , W. Lu , T. Dai , and L. Wang , “Our Experience With Propranolol for Infantile Hemangioma,” Skin Research and Technology 29, no. 4 (2023): e13310, 10.1111/srt.13310. 37113082 PMC10234167

[37] A. Morimoto , M. Ozeki , S. Sasaki , N. Baba , Y. Kuwano , and T. Kaneko , “Severe Hypoglycemia in Propranolol Treatment for Infantile Hemangiomas,” Pediatrics International 64, no. 1 (2022): e15278, 10.1111/ped.15278. 35972062 PMC9541900

[38] E. Fernández Faith , S. Shah , P. M. Witman , et al., “Clinical Features, Prognostic Factors, and Treatment Interventions for Ulceration in Patients With Infantile Hemangioma,” JAMA Dermatology 157, no. 5 (2021): 566–572, 10.1001/jamadermatol.2021.0469. 33787840 PMC8014192

[39] K. Han , J. Wei , H. Zheng , et al., “Efficacy and Safety of Oral Propranolol or Topical Timolol for the Treatment of Superficial Infantile Hemangiomas,” Journal of Craniofacial Surgery 35 (2024): e254–e257, 10.1097/SCS.0000000000010001. 38345941

[40] M. Xia , K. Ding , Y. Ji , et al., “The Timing and Safety of Topical Timolol Treatment for Superficial Infantile Hemangioma: A Retrospective Cohort Study,” European Journal of Pediatrics 184, no. 2 (2025): 151, 10.1007/s00431-025-05983-3. 39853550 PMC11761464

[41] A. Mazhar , A. Naureen , Y. A. Mallick , and L. Samad , “Efficacy of 0.5% Timolol Maleate Gel in the Management of Infantile Hemangiomas. Pak,” Journal of Medical Sciences 40, no. Suppl 2 (2024): S75–S79, 10.12669/pjms.40.2(ICON)0.8983. PMC10844907 38328662

[42] F. Z. Muñoz‐Garza , M. Ríos , E. Roé‐Crespo , et al., “Efficacy and Safety of Topical Timolol for the Treatment of Infantile Hemangioma in the Early Proliferative Stage: A Randomized Clinical Trial,” JAMA Dermatology 157, no. 5 (2021): 583–587, 10.1001/jamadermatol.2021.0596. 33825828 PMC8027942

[43] T. Qiu , S. Dai , X. Jiang , et al., “Efficacy and Safety of Propranolol vs. Atenolol in Infants With Problematic Infantile Hemangiomas: A Randomized Clinical Trial. JAMA,” Otolaryngology and Head and Neck Surgery 147, no. 7 (2021): 599–607, 10.1001/jamaoto.2021.0454. PMC8050788 33856430

[44] S. A. Pattanshetti , V. M. Mahalmani , P. Sarma , et al., “Oral Atenolol Versus Propranolol in the Treatment of Infantile Hemangioma: A Systematic Review and Meta‐Analysis,” Journal of Indian Association of Pediatric Surgeons 27, no. 3 (2022): 279–286, 10.4103/jiaps.jiaps_3_21. 35733601 PMC9208683

[45] M. M. Hermans , S. G. M. A. Pasmans , P. C. J. de Laat , et al., “Propranolol or Atenolol for the Management of Infantile Hemangioma: Implications for Long‐Term Health,” JAAD Int 11 (2023): 137–139, 10.1016/j.jdin.2023.02.001. 37128266 PMC10148149

[46] M. M. Hermans , R. Schappin , P. C. J. de Laat , et al., “Mental Health of School‐Aged Children Treated With Propranolol or Atenolol for Infantile Hemangioma and Their Parents,” Dermatology 240, no. 2 (2024): 216–225, 10.1159/000536144. 38228125 PMC10997238

[47] Y. Ji , S. Chen , K. Yang , et al., “Efficacy and Safety of Propranolol vs. Atenolol in Infants With Problematic Infantile Hemangiomas: A Randomized Clinical Trial. JAMA,” Otolaryngology and Head and Neck Surgery 147, no. 7 (2021): 599–607, 10.1001/jamaoto.2021.0454. PMC8050788 33856430

[48] S. Taddei , N. Tsabedze , and R. S. Tan , “β‐Blockers Are Not All the Same: Pharmacologic Similarities and Differences, Potential Combinations and Clinical Implications,” Current Medical Research and Opinion 40, no. Suppl 1 (2024): 15–23, 10.1080/03007995.2024.2318058. 38597065

[49] D. M. Adams and K. W. Ricci , “Vascular Anomalies: Diagnosis of Complicated Anomalies and New Medical Treatment Options,” Hematology/Oncology Clinics of North America 33, no. 3 (2019): 455–470, 10.1016/j.hoc.2019.01.011. 31030813

[50] G. Wang , W. Lu , Y. Zhu , C. Wang , and X. Yang , “Effectiveness and Safety of Sirolimus in the Treatment of Venous Malformations: A Meta‐Analysis of Prospective Studies,” Journal of Vascular Surgery. Venous and Lymphatic Disorders 13, no. 6 (2025): 102284, 10.1016/j.jvsv.2025.102284. 40619100 PMC12340385

[51] S. Faes , T. Santoro , N. Demartines , and O. Dormond , “Evolving Significance and Future Relevance of Anti‐Angiogenic Activity of mTOR Inhibitors in Cancer Therapy,” Cancers (Basel) 9, no. 11 (2017): 152, 10.3390/cancers9110152. 29104248 PMC5704170

[52] K. M. Dodd , J. Yang , M. H. Shen , J. R. Sampson , and A. R. Tee , “mTORC1 Drives HIF‐1α and VEGF‐A Signalling via Multiple Mechanisms Involving 4E‐BP1, S6K1 and STAT3,” Oncogene 34, no. 17 (2015): 2239–2250, 10.1038/onc.2014.164. 24931163 PMC4172452

[53] S. Chouhan , A. Kumar , V. Piprode , A. Dasgupta , S. Singh , and A. Khalique , “Regulatory‐Associated Protein of mTOR‐Mediated Signaling: A Nexus Between Tumorigenesis and Disease,” Targets 2, no. 4 (2024): 341–371, 10.3390/targets2040020.

[54] D. D. Kim , D. M. Kleinman , T. Kanetaka , et al., “Rapamycin Inhibits VEGF‐Induced Microvascular Hyperpermeability in Vivo,” Microcirculation 17, no. 2 (2010): 128–136, 10.1111/j.1549-8719.2009.00012.x. 20163539 PMC2925471

[55] B. Asani , J. Siedlecki , C. Wertheimer , et al., “Anti‐Angiogenic Properties of Rapamycin on Human Retinal Pericytes in an in Vitro Model of Neovascular AMD via Inhibition of the mTOR Pathway,” BMC Ophthalmology 22, no. 1 (2022): 138, 10.1186/s12886-022-02334-w. 35337287 PMC8957126

[56] D. M. Adams , C. C. Trenor, 3rd , A. M. Hammill , et al., “Efficacy and Safety of Sirolimus in the Treatment of Complicated Vascular Anomalies,” Pediatrics 137, no. 2 (2016): e20153257, 10.1542/peds.2015-3257. 26783326 PMC4732362

[57] E. Seront , A. Van Damme , C. Legrand , et al., “Preliminary Results of the European Multicentric Phase III Trial Regarding Sirolimus in Slow‐Flow Vascular Malformations,” JCI Insight 8, no. 21 (2023): e173095, 10.1172/jci.insight.173095. 37937645 PMC10721262

[58] A. M. Saibene , C. Rosso , G. Felisati , et al., “Sirolimus Treatment for Paediatric Head and Neck Lymphatic Malformations: A Systematic Review,” European Archives of Oto‐Rhino‐Laryngology 280, no. 8 (2023): 3529–3540, 10.1007/s00405-023-07991-1. 37115326 PMC10313583

[59] S. Wiegand , A. Dietz , and G. Wichmann , “Efficacy of Sirolimus in Children With Lymphatic Malformations of the Head and Neck,” European Archives of Oto‐Rhino‐Laryngology 279, no. 8 (2022): 3801–3810, 10.1007/s00405-022-07378-8. 35526176 PMC9249683

[60] A. Maruani , E. Tavernier , O. Boccara , et al., “Sirolimus (Rapamycin) for Slow‐Flow Malformations in Children: The Observational‐Phase Randomized Clinical PERFORMUS Trial,” JAMA Dermatology 157, no. 11 (2021): 1289–1298, 10.1001/jamadermatol.2021.3459. 34524406 PMC8444064

[61] V. E. M. Harbers , L. G. J. M. Zwerink , G. A. Rongen , et al., “Clinical Differences in Sirolimus Treatment With Low Target Levels Between Children and Adults With Vascular Malformations—A Nationwide Trial,” Clinical and Translational Science 16, no. 5 (2023): 781–796, 10.1111/cts.13488. 36824030 PMC10176016

[62] N. Zheng , X. Ding , and R. Jahan , “Low Concentration of Rapamycin Inhibits Hemangioma Endothelial Cell Proliferation, Migration, and Vascular Tumor Formation in Mice,” Current Therapeutic Research, Clinical and Experimental 76 (2014): 99–103, 10.1016/j.curtheres.2014.09.004. 25408787 PMC4229512

[63] S. Greenberger , S. Yuan , L. A. Walsh , et al., “Rapamycin Suppresses Self‐Renewal and Vasculogenic Potential of Stem Cells Isolated From Infantile Hemangioma,” Journal of Investigative Dermatology 131, no. 12 (2011): 2467–2476, 10.1038/jid.2011.300. 21938011 PMC3213330

[64] M. Dodds , M. Tollefson , L. Castelo‐Soccio , et al., “Treatment of Superficial Vascular Anomalies With Topical Sirolimus: A Multicenter Case Series,” Pediatric Dermatology 37, no. 2 (2020): 272–277, 10.1111/pde.14104. 31957126

[65] O. Tezol , M. Alakaya , B. Gundogan , and E. C. Citak , “Sirolimus for the Treatment of Benign Vascular Anomalies in Children: A Single Centre Experience,” Hong Kong J Paediatr (New Series) 28 (2023): 20–26.

[66] V. Dekeuleneer , A. Van Damme , D. Dumitriu , et al., “Traitement d'un hémangiome infantile résistant au propranolol par une courte cure de sirolimus,” Annales de Dermatologie et de Vénéréologie 3, no. 8 Suppl 1 (2023): A320–A321.

[67] B. Abtahi‐Naeini , F. Ahmadinia , F. Ghorbali , and E. Foroughi , “A Systematic Review of the Efficacy and Safety of Sirolimus (Rapamycin) in the Management of Infantile Hemangioma,” J Res Pharm Pract 14, no. 4 (2025): 127–133, 10.4103/jrpp.jrpp_58_25. 41368601 PMC12684998

[68] M. Aisyi , A. H. Syarif , D. A. Mukarramah , R. Hermawan , and D. Iriani , “Everolimus for the Treatment of Epithelioid Hemangioma: A Case Report,” Med J Indones 30, no. 4 (2021): 301–305, https://mji.ui.ac.id/journal/index.php/mji/article/view/5036.

[69] R. Xie , Z. Taohuang , R. Kieran , L. Chen , Y. Li , and M. Zhao , “Everolimus and Sunitinib Potentially Work as Therapeutic Drugs for Infantile Hemangiomas,” Pediatric Research 98 (2025): 2374–2384, 10.1038/s41390-025-04028-7. 40188217 PMC12811115

[70] T. Wilhoit , J. M. Patrick , and M. B. May , “Alpelisib: A Novel Therapy for Patients With PIK3CA‐Mutated Metastatic Breast Cancer,” Journal of the Advanced Practitioner in Oncology 11, no. 7 (2020): 768–775, 10.6004/jadpro.2020.11.7.9. 33575071 PMC7646628

[71] Q. Venot , T. Blanc , S. H. Rabia , et al., “Targeted Therapy in Patients With PIK3CA‐Related Overgrowth Syndrome,” Nature 558, no. 7711 (2018): 540–546, 10.1038/s41586-018-0217-9. 29899452 PMC7610773

[72] L. Zerbib , S. Ladraa , A. Fraissenon , et al., “Targeted Therapy for Capillary‐Venous Malformations,” Signal Transduction and Targeted Therapy 9, no. 1 (2024): 146, 10.1038/s41392-024-01862-9. 38880808 PMC11180659

[73] P. Kobialka , H. Sabata , O. Vilalta , et al., “The Onset of PI3K‐Related Vascular Malformations Occurs During Angiogenesis and Is Prevented by the AKT Inhibitor Miransertib,” EMBO Molecular Medicine 14, no. 7 (2022): e15619, 10.15252/emmm.202115619. 35695059 PMC9260211

[74] C. Ranieri , S. Di Tommaso , D. C. Loconte , et al., “In Vitro Efficacy of ARQ 092, an Allosteric AKT Inhibitor, on Primary Fibroblast Cells Derived From Patients With PIK3CA‐Related Overgrowth Spectrum (PROS),” Neurogenetics 19, no. 2 (2018): 77–91. 29549527 10.1007/s10048-018-0540-1 PMC5956072

[75] K. M. Keppler‐Noreuil , J. C. Sapp , M. J. Lindhurst , et al., “Pharmacodynamic Study of Miransertib in Individuals With Proteus Syndrome,” American Journal of Human Genetics 104, no. 3 (2019): 484–491. 30803705 10.1016/j.ajhg.2019.01.015 PMC6407523

[76] K. Forde , N. Resta , C. Ranieri , et al., “Clinical Experience With the AKT1 Inhibitor Miransertib in Two Children With PIK3CA‐Related Overgrowth Syndrome,” Orphanet Journal of Rare Diseases 16, no. 1 (2021): 109. 33639990 10.1186/s13023-021-01745-0 PMC7913425

[77] E. Jonasch , E. Hasanov , P. G. Corn , et al., “A Randomized Phase 2 Study of MK‐2206 Versus Everolimus in Refractory Renal Cell Carcinoma,” Annals of Oncology 28, no. 4 (2017): 804–808, 10.1093/annonc/mdw676. 28049139 PMC5834088

[78] S. Faes , N. Demartines , and O. Dormond , “Resistance to mTORC1 Inhibitors in Cancer Therapy: From Kinase Mutations to Intratumoral Heterogeneity of Kinase Activity,” Oxidative Medicine and Cellular Longevity 2017 (2017): 1726078, 10.1155/2017/1726078. 28280521 PMC5322438

[79] E. J. Walker , H. Su , F. Shen , et al., “Bevacizumab Attenuates VEGF‐Induced Angiogenesis and Vascular Malformations in the Adult Mouse Brain,” Stroke 43, no. 7 (2012): 1925–1930, 10.1161/STROKEAHA.111.647982. 22569934 PMC3404823

[80] H. H. Sabry , N. E. Sorour , and E. M. Akl , “Intralesional Injection of Bevacizumab Versus Triamcinolone Acetonide in Infantile Hemangioma,” Journal of Dermatological Treatment 31, no. 3 (2020): 279–284, 10.1080/09546634.2019.1590521. 30835573

[81] S. Greenberger , E. Boscolo , I. Adini , J. B. Mulliken , and J. Bischoff , “Corticosteroid Suppression of VEGF‐A in Infantile Hemangioma‐Derived Stem Cells,” New England Journal of Medicine 362, no. 11 (2010): 1005–1013, 10.1056/NEJMoa0903036. 20237346 PMC2845924

[82] X. Peng , X. Huang , T. B. Lulu , et al., “A Novel Pan‐PI3K Inhibitor KTC1101 Synergizes With Anti‐PD‐1 Therapy by Targeting Tumor Suppression and Immune Activation,” Molecular Cancer 23 (2024): 54, 10.1186/s12943-024-01978-0. 38486218 PMC10938783

[83] R. R. McKay , G. De Velasco , L. Werner , et al., “A Phase 1 Study of Buparlisib and Bevacizumab in Patients With Metastatic Renal Cell Carcinoma Progressing on VEGF‐Targeted Therapies,” Cancer 122, no. 15 (2016): 2389–2398, 10.1002/cncr.30056. 27198170 PMC5567751

[84] H. L. Nguyen , L. M. Boon , and M. Vikkula , “Trametinib as a Promising Therapeutic Option in Alleviating Vascular Defects in an Endothelial KRAS‐Induced Mouse Model,” Human Molecular Genetics 32, no. 2 (2023): 276–289, 10.1093/hmg/ddac169. 35972810

[85] J. B. Foster , D. Li , M. E. March , et al., “Kaposiform Lymphangiomatosis Effectively Treated With MEK Inhibition,” EMBO Molecular Medicine 12, no. 10 (2020): e12324, 10.15252/emmm.202012324. 32894644 PMC7539180

[86] S. Watanabe , M. Manabe , M. Miyata , A. Naoe , and T. Suzuki , “A Case of Neonate Effectively Treated With Everolimus for Giant Hepatic Hemangioma Complicated With Congenital Duodenal Atresia and Kasabach‐Merritt Syndrome,” Journal of Neonatal‐Perinatal Medicine 14, no. 3 (2021): 437–440, 10.3233/NPM-200504. 33325401

[87] G. Andelfinger , C. Marquis , M. J. Raboisson , et al., “Hypertrophic Cardiomyopathy in Noonan Syndrome Treated by MEK‐Inhibition,” Journal of the American College of Cardiology 73, no. 17 (2019): 2237–2239, 10.1016/j.jacc.2019.01.066. 31047013 PMC6916648

[88] A. Fraissenon , C. Bayard , G. Morin , et al., “Sotorasib for Vascular Malformations Associated With KRAS G12C Mutation,” New England Journal of Medicine 391, no. 4 (2024): 334–342, 10.1056/NEJMoa2309160. 39018528

[89] J. Dong , J. Cui , X. Shi , T. Wang , and S. Liu , “Itraconazole Inhibits Proliferation, Induces Apoptosis, and Reduces Angiogenesis of Hemangioma Endothelial Cells by Downregulating the Hedgehog Signaling Pathway,” Heliyon 9, no. 9 (2023): e19244, 10.1016/j.heliyon.2023.e19244. 37674841 PMC10477473

[90] H. Bessar , A. H. Kandil , N. M. Nasr , and F. Khattab , “Itraconazole Versus Propranolol: Therapeutic and Pharmacologic Effect on Serum Angiopoietin‐2 in Patients With Infantile Hemangioma,” Journal of Dermatological Treatment 33, no. 1 (2022): 105–110, 10.1080/09546634.2019.1687822. 31668109

[91] X. He , X. Ran , D. Liang , H. Fan , and Y. Ran , “Itraconazole Oral Solution for Infantile Complicated Hemangioma With Double Lesions on the Skin and One Inside the Liver,” Clinical, Cosmetic and Investigational Dermatology 17 (2024): 1217–1226, 10.2147/CCID.S462665. 38803817 PMC11129757

[92] W. Lu , Z. Yang , M. Wang , Y. Zhang , Z. Qi , and X. Yang , “Identification of Potential Therapeutics for Infantile Hemangioma via in Silico Investigation and in Vitro Validation,” Drug Design, Development and Therapy 18 (2024): 4065–4088, 10.2147/DDDT.S460575. 39286286 PMC11404501

[93] D. Li , S. E. Sheppard , M. E. March , et al., “Genomic Profiling Informs Diagnoses and Treatment in Vascular Anomalies,” Nature Medicine 29, no. 6 (2023): 1530–1539, 10.1038/s41591-023-02364-x. PMC11184491 37264205

